# Guidezilla™ guide extension catheter I for transradial coronary intervention

**DOI:** 10.3389/fcvm.2022.931373

**Published:** 2022-08-17

**Authors:** Xinjun Lei, Qi Liang, Yuan Fang, Yihui Xiao, Dongqi Wang, Maozhi Dong, Jiancheng Li, Ting Yu

**Affiliations:** ^1^Department of Cardiovascular Medicine, The First Affiliated Hospital of Xi’an Jiaotong University, Xi’an, China; ^2^Department of Cardiovascular Medicine, Shangnan People’s Hospital, Shangluo, China; ^3^Department of Cardiovascular Medicine, Shangluo Central Hospital, Shangluo, China

**Keywords:** Guidezilla™, complex coronary lesions, transradial, SYNTAX score, percutaneous coronary intervention, case series

## Abstract

**Background:**

Percutaneous coronary intervention (PCI) is the preferred treatment method for coronary artery diseases (CAD). This study aimed to evaluate the effectiveness and complications of the Guidezilla™ guide extension catheter I (GGEC I) in transradial coronary intervention (TRI).

**Methods:**

This case series study included patients with CAD who underwent TRI using the GGEC I between August 2016 and January 2019 at the First Affiliated Hospital of Xi’an Jiaotong University.

**Results:**

A total of 221 patients aged 65.1 ± 9.26 years were included. Coronary angiography results indicated that most patients (77.8%) had triple-vessel lesions, including 47.5% with chronic total occlusion (CTO). A total of 237 target lesions were treated, most being type C lesions (95.8%). The most common indication for GGEC I use was heavy calcification (67%), followed by extreme tortuosity (12.2%), extreme tortuosity and heavy calcification (10.9%), distally located lesion (4.5%), picking up the retrograde wire (3.2%), anomalous vessel origin (1.8%), and releasing the burr incarceration (0.4%). The mean operation time was 58 min, and the overall success rate was 94.1%. Four patients received a drug-coated balloon. No significant differences were found in operation time and success rate among the low (<23), intermediate (23–32), and severe (>32) CAD groups based on SYNTAX score stratification (*P* > 0.05). Two subacute thrombosis cases each were reported perioperatively, during hospitalization, and at the 1-month follow-up.

**Conclusion:**

The GGEC I might have advantages for TRI and is unaffected by SYNTAX score stratification.

## Introduction

Percutaneous coronary intervention (PCI) is the preferred treatment method for coronary artery diseases (CAD). Most complex coronary artery lesions can be treated owing to the continuous refinements of the hardware armamentarium and improvements of interventional technologies. Notably, some complex target lesions are difficult to reach using the current tools because of the inadequate backup support of the guiding catheter (1), especially in highly calcified and tortuous vessels (2). This is one of the main reasons for the failure of PCI in complex CAD.

Recently, transradial coronary intervention (TRI) has gained popularity in the treatment of CAD because of advantages such as low incidence rates of access site bleeding and vascular complications, early ambulation, improved patient comfort, and short hospital stay (3, 4). Furthermore, several clinical trials (4, 5) and meta-analyses (6) associated TRI with higher procedural success rates, lower mortality rates, and comparable main adverse cardiovascular and cerebrovascular event (MACCE) rates compared with TFI. Therefore, substantial research efforts have been directed toward strengthening the backup support of the original guiding catheter and guidewire for the successful management of complex coronary lesions. An extra support guidewire, buddy wire, anchoring balloon, and guiding catheter deep intubation techniques are standard for strengthening backup support (2, 7, 8). Nevertheless, these solutions carry risks such as wire entanglement, anchoring vascular injury, coronary artery dissection, iatrogenic aortocoronary dissection (IACD), and even coronary artery perforation (9).

Few studies have examined the Guidezilla™ guide extension catheter (GGEC) as an alternative for treating complex coronary artery lesions with TRI. The GGEC is a unique, rapid-exchange, mother-in-child catheter that increases the backup support for the guiding catheter by facilitating deep coronary intubation and coaxial alignment, and also enables a smooth delivery of the interventional device to the target lesion for successful completion of PCI (10, 11) and TRI (12–14).

The European and American guidelines state that the anatomical SYNTAX score is an essential tool that could help clinicians choose the most appropriate revascularization strategy for patients with complex CAD-PCI or coronary artery bypass graft (CABG) surgery (15, 16). Based on the anatomical severity of CAD, the patients can be categorized as low (<23), intermediate (23–32), or severe (>32) category according to the SYNTAX score. It was found that low- and intermediate-category patients have similar long-term outcomes regardless of the revascularization strategy implemented (15, 16). In contrast, CABG yields better outcomes for severe cases than PCI (17). Nevertheless, the SYNTAX scoring system lacks an individualized approach and clinical variables to guide the choice of the revascularization strategy accurately.

On the other hand, the SYNTAX II score contains eight predictors: anatomical SYNTAX score, age, creatinine clearance, left ventricular ejection fraction (LVEF), unprotected left main coronary artery (ULMCA) disease, peripheral vascular disease, female sex, and chronic obstructive pulmonary disease (COPD) (18, 19). It can significantly predict the difference in 4-year mortality between patients who underwent CABG and PCI. As such, this version is better in assisting the choice of CABG or PCI for patients with complex CAD compared with the original SYNTAX score. Although several observational studies on PCI for CAD provided evidence for the benefits and low complication rates of the GGEC, (10–14) no research has investigated the impact of the SYNTAX score on treatment outcomes.

Therefore, this study aimed to evaluate the effectiveness and complications of the GGEC I in TRI for patients stratified according to the SYNTAX score.

## Materials and methods

### Study design and participants

This case series study included patients with CAD who underwent TRI using GGEC I between August 2016 and January 2019 at the First Affiliated Hospital of Xi’an Jiaotong University, Xi’an, China. This study was approved by the Ethics Committee for Human Study of the First Affiliated Hospital of Xi’an Jiaotong University. The study was conducted in compliance with the ethical principles of the Declaration of Helsinki. The requirement for informed consent was waived by the committee.

### Data collection and definition

Data were collected from medical records, including age, sex, clinical presentation, coronary angiography indication, target vessel and character of the lesion, type of guiding catheter, guidewire, balloon, stent, operative time, surgical outcome, dissection, stent dislodgement, shaft breakage, in-hospital complications, and at 1-month follow-up after TRI.

The target lesions were classified as type A, B1, B2, or C based on the American Heart Association/American College of Cardiology (AHA/ACC) criteria using variables such as length, angulation, tortuosity, calcification, and chronicity (20). Angulation was estimated by recording the angle formed between the proximal and distal vessel axes (≥45° = moderate; ≥90° = severe). A tortuous lesion was defined as having at least three ≥ 45° bends in the vessel direction along the main trunk during the diastolic period. Calcification was determined based on the density of the vessel wall before injection of the contrast agent.

The SYNTAX score was used to assess the complexity of CAD and assist clinicians in choosing the most appropriate revascularization strategy for patients (20). First, the SYNTAX I score was evaluated for each patient based on coronary angiography results by two experienced interventional cardiologists. Then, the SYNTAX II score was calculated by evaluating clinical variables (age, creatinine clearance, LVEF, ULMCA disease, peripheral vascular disease, female sex, and COPD). These variables can be automatically calculated on the SYNTAX website at the time. Based on the SYNTAX I score, the patients were categorized as low (<23), intermediate (23–32), or severe (>32) CAD cases.

**FIGURE 1:**
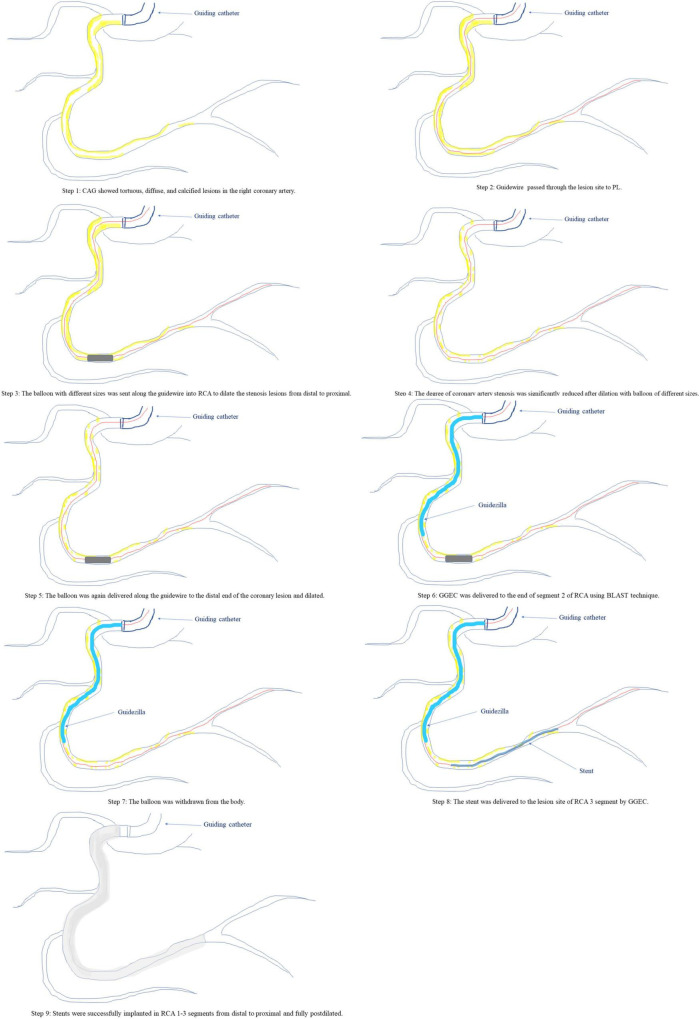
A schematic diagram of the GGEC I in complex PCI.

The GGEC I (Boston Scientific, Natick, MA, United States) is a 145-cm single-lumen rapid exchange catheter compatible with a 6F guiding catheter with an inner diameter of 0.057 inches (1.45 mm) and an outer diameter of 0.066 inches (1.68 mm). The guiding catheter is a 120-cm stainless steel hypo-tube with a 25-cm special wire-braided mesh/polymer structure. In the patients included in this study, the GGEC I was mainly used as a salvage treatment to create a smooth pathway for delivering a balloon or stent to the target lesion after high-pressure balloon pre-dilatation, without replacing the original guiding catheter. The specific application indications were: (1) anomalous origin or angulated take-off of native coronary arteries; (2) extremely tortuous vessel; (3) heavy calcification; (4) distally located lesion; (5) picking up the retrograde wire during a CTO intervention; (6) releasing the burr incarceration. In the presence of multiple indications, the main reason requiring GGEC I use was listed as the primary indication. A deep GGEC I intubation was defined as more than 20 mm depth. A schematic diagram of GGEC I use in complex PCI is shown in Figure 1. In this study, experienced interventional cardiologists performed TRI with the GGEC I according to standard clinical protocols. The other courses of treatment were decided by the attending physician and the consulting operator.

Effectiveness was determined by the success of implanting a stent or drug-coated balloon (DCB) angioplasty in the targeted lesion area with residual stenosis of less than 20% and TIMI 3 flow. Complications were defined as procedure-related complications (e.g., dissection, perforation, stent stripping or dislodgement, shaft breakage, and acute thrombosis) and major clinical events (e.g., intractable angina, recurrence of myocardial infarction, repeated revascularization, and all-cause death) during the hospital stay and a 1-month follow-up.

### Statistical analysis

Statistical analyses were performed using SPSS 22.0 (IBM, Armonk, NY, United States). Continuous variables were expressed as median [interquartile range (IQR)] and compared by the Wilcoxon signed-rank test. Categorical data were presented as n (%) and compared by the chi-square (χ^2^) test. All the contributing authors guaranteed the reliability, interpretation, and lack of bias for all the investigated aspects of the study. *P* < 0.05 was considered statistically significant.

## Results

The detailed baseline characteristics of the 221 patients included in the study are presented in Table 1. The mean age was 65.1 ± 9.26 years. Older patients (over the age of 65) accounted for 57% of the population (*n* = 126). The number of male patients (*n* = 179, 81%) was higher than that of female patients (*n* = 42, 19%). There were 157 patients with hypertension (71%) and 114 smokers (51.6%). There were 110 patients with elevated low-density lipoprotein cholesterol (LDL-C; 49.8%) and 78 with diabetes mellitus (35.3%). The most common clinical presentation was unstable angina (UA; *n* = 136, 61.5%), followed by chronic stable angina (CSA; *n* = 36, 16.3%), non-ST-segment elevation myocardial infarction (NSTEMI; *n* = 27, 12.2%), and ST-segment elevation myocardial infarction (STEMI; *n* = 22, 10.0%) (Table 1).

**TABLE 1:** Patient baseline characteristics (*n* = 221).

Index	Value
Age (years)	65.1 ± 9.26
<55, *n* (%)	31 (14.0)
55–65, *n* (%)	64 (29.0)
>65, *n* (%)	126 (57.0)
Male, *n* (%)	179 (81.0)
Smoking, *n* (%)	114 (51.6)
Hypertension, *n* (%)	157 (71.0)
Diabetes mellitus, *n* (%)	78 (35.3)
LDL-C (mmol⋅L^–1^)	1.96 ± 0.79
LDL-C > 1.8, *n* (%)	110 (49.8)
HDL-C (mmol⋅L^–1^)	0.88 ± 0.19
LP(a) (mg⋅L^–1^)	236.06 ± 164
TG (mmol⋅L^–1^)	1.60 ± 1.28
HCY (μmol⋅L^–1^)	23.24 ± 19.10
BUN (mmol⋅L^–1^)	4.47 ± 3.89
Cr, M (P25, P75) (μmol⋅L^–1^)	67 (56, 80)
eGFR, M (P25, P75) (mL⋅m^–1^⋅1.73 m^–1^)	95.84 (83.35, 104.37)
NT-ProBNP, M (P25, P75) (pg⋅mL^–1^)	425.30 (128.73, 1397.0)
Left ventricular ejection fraction (%)	57.02 ± 11.91
**Angiography indication**	
UA, *n* (%)	136 (61.5%)
CSA, *n* (%)	36 (16.3%)
NSTEMI, *n* (%)	27 (12.2%)
STEMI, *n* (%)	22 (10.0%)

LDL-C, low-density lipoprotein cholesterol (according to existing international and domestic guidelines, LDL-C levels in patients with coronary heart disease should be kept below 1.8 mmol⋅L^–1^); UA, unstable angina; CSA, chronic stable angina; NSTEMI, non-ST-segment elevation myocardial infarction; STEMI, ST-segment elevation myocardial infarction.

Most patients suffered from triple-vessel lesions (*n* = 172, 77.8%), including 35 (15.8%) with left main CAD (LMCAD) and 105 (47.5%) with CTO. According to SYNTAX I scores, the percentages of low (<23), intermediate (23–32), and severe (>32) CAD cases were 9.9% (*n* = 22), 25.8% (*n* = 57), and 64.3% (*n* = 142), respectively. The average SYNTAX II score for PCI was 32.7 ± 7.9, with an estimated 4-year mortality rate of 10.4 ± 8.8%, while the average SYNTAX II score for CABG was 24.2 ± 7.6, with a much lower estimated 4-year mortality rate of 5.1 ± 3.3%. Theoretically, a clear majority of patients in this study should have received CABG instead of TRI. Although 49.3% (*n* = 109) of patients with complex CAD were recommended to undergo CABG, they opted for TRI after full consideration (Table 2).

**TABLE 2:** Characteristics of coronary artery disease and SYNTAX score of patients who underwent TRI using the GGEC I (*n* = 221).

Types of coronary artery disease	Results
Single-vessel lesion, *n* (%)	9 (4.1)
Double-vessel lesion, *n* (%)	40 (18.1)
Triple-vessel lesion, *n* (%)	172 (77.8)
LMCAD, *n* (%)	35 (15.8)
**CTO:**	
Single-vessel CTO, *n* (%)	89 (40.3)
Double-vessel CTO, *n* (%)	14 (6.3)
Triple-vessel CTO, *n* (%)	2 (0.9)
**SYNTAX I Score:**	
<22, *n* (%) Average	22 (9.9) 18.23 ± 2.57
23–32, *n* (%) Average	57 (25.8) 28.05 ± 20.87
≥33, *n* (%) Average	142 (64.3) 44.11 ± 8.98
**SYNTAX II Score:**	
PCI	32.66 ± 70.88
4 years mortality (%)	10.44 ± 8.81
CABG	24.17 ± 7.55
4 years mortality (%)	5.14 ± 3.34
**Treatment recommendation:**	
CABG or PCI, *n* (%)	112 (50.7)
CABG, *n* (%)	109 (49.3)

**FIGURE 2:**
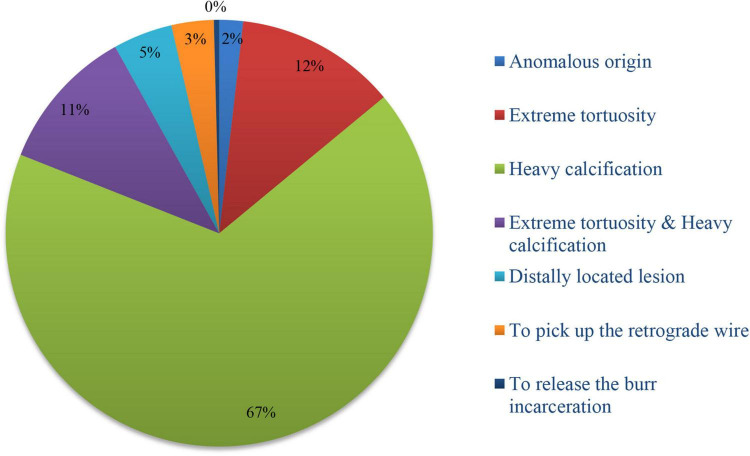
Indications for GGEC I use in TRI.

**FIGURE 3:**
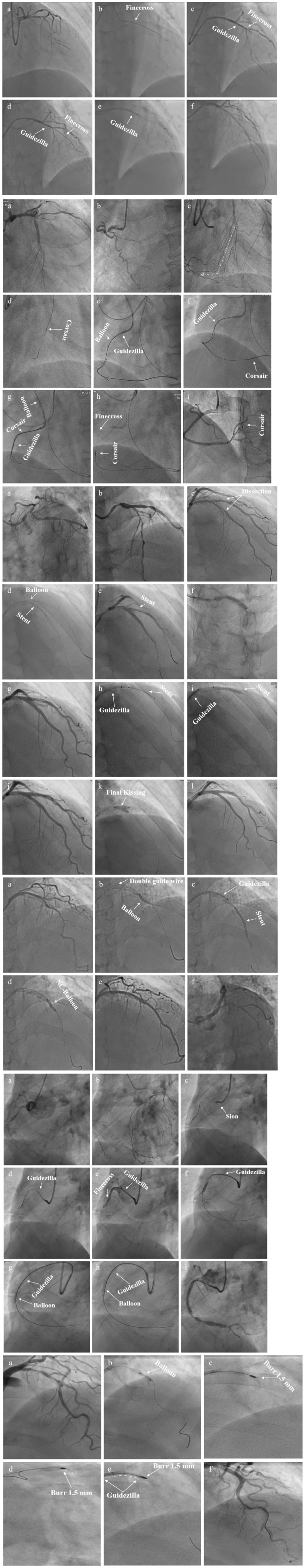
Typical examples of TRI using the GGEC I in different situation (**Case 1–6**). **Case 1**: an example of percutaneous coronary intervention (PCI) using the Guidezilla™ guide extension catheter I (GGEC I) in left ascending artery chronic total occlusion (LAD CTO) using the antegrade wire approach. A 6F BL3.5 guiding catheter angiography showed the LAD 7-segment CTO **(a)**. Miracle 6.0 was first selected under the support of a Finecross microcatheter, but failed due to insufficient support of the guiding catheter **(b)**. GGEC I was sent along Miracle 6.0 to the LAD 6-segment and then the microcatheter was used to control Miracle 6.0 to smoothly penetrate the proximal fibrous cap of the occlusive disease **(c)**. Miracle 6.0 was then carefully manipulated into the true lumen of the occluded distal blood vessel to the end of the LAD **(d)**. Then, Miracle 6.0 was replaced with Sion via the Finecross microcatheter **(e)**. Finally, the stent was successfully implanted after pre-expansion with balloons of different specifications **(f)**. **Case 2**: An example of PCI using the GGEC I in right coronary artery (RCA) CTO with retrograde wire approach. TIG angiography showing the LAD 7-segment CTO **(a)** and RCA 1-segment CTO **(b)**. First, the antegrade wire approach was used to open the LAD, and then the retrograde wire approach was used to open the RCA CTO (**c**, the white dotted line shows the retrograde pathway used in the operation). Under the support of the Corsair microcatheter, Sion was carefully manipulated through the S1 collateral branch **(d)** to advance the Corsair microcatheter to the RCA 3-segment along the guidewire, and changed to the UB 3 via the microcatheter; then, UB 3 was directed to the RCA 2-segment through the occluded vessel distal segment. Next, Miracle 6.0 was forwardly manipulated to penetrate the proximal fibrous cap of the occlusive lesion to the RCA 3-segment and the GGEC I was sent along Miracle 6.0 to the RCA1 segment. Then, the Reverse-Controlled Antegrade And Retrograde Subintimal Tracking (R-CART) technique was initiated **(e)**, using Active Greeting Technique (AGT) to reversely manipulate Fielder XT into GGEC I **(f)**. Fielder XT was pushed further forward into 6F SAL 1.0, then anchored with a balloon to push Corsair into 6F SAL 1.0 forcefully **(g)**. The Rendezvous technique failed, and Sion was reverse manipulated into the forward Finecross microcatheter through the Corsair microcatheter, and the Finecross microcatheter was pushed forward to the posterior branches of left ventricular (PL) along Sion while withdrawing the Corsair microcatheter backward **(h)**. Stents were implanted after dilating the occluded blood vessel with predilated balloons of different sizes along the guidewire after the Finecross microcatheter was removed **(i)**. **Case 3**: An example of PCI using GGEC I in both LM and LAD bifurcation lesions. 6F JL3.5 angiography showed approximately 80% stenosis of the LM end, 50-80% stenosis of the LAD 6-7 segment, and subtotal occlusion in the proximal segment of D1 **(a,b)**. The two Runthroughs were carefully manipulated to enter the ends of the LAD and D1, respectively, and narrow lesions were expanded with a balloon, resulting in residual stenosis of approximately 50% in the LM end, and severe dissection of LAD7 and proximal D1 with thrombolysis in myocardial infarction (TIMI) flow grade 3 as seen by angiography **(c)**. The Crossover strategy was used to treat LM bifurcation lesions, and the inverse mini-crush technology was used to treat LAD and D1 bifurcation lesions **(d)**. However, after repeated attempts, it was difficult for the stent to enter D1 and completely cover the lesion **(e)**, so a stent was immediately implanted in LM-LAD **(f)**. Angiography showed that the stent was fully expanded, with a blood flow of TIMI level 3 **(g)**. Next, a GGEC I was sent along the guidewire to the opening of D1, and a stent successfully sent to D1 via the GGEC I and completely covered the lesion **(h)**. After withdrawing the GGEC I to the LAD opening, the stent was successfully released in the D1 **(i)**. Angiography showed that the stent was fully expanded and the blood flow was TIMI level 3 **(j)**. Finally, by rewiring the Runthrough into the LAD and completing the Final Kissing step **(k)**, the operation was a success **(l)**. **Case 4**: An example of PCI using the GGEC I in LAD with extreme tortuosity and severely calcified lesions. 6F EBU 3.75 angiography showed extreme tortuosity and heavy calcification in LAD 6–8 segments with approximately 80% stenosis **(a)**. Two Runthroughs were manipulated to reach the end of the LAD through the stenosis, and Sprinter 2.0 × 15 mm and NC Sprinter 2.5 × 15 mm were pushed into place in turn with difficulty, under the support of a double guidewire, which was used to expand the stenosis under high pressure (20–24 atm) **(b)**. Then, three stents were implanted via the GGEC I from distal to proximal LAD 6–8 **(c)**. Finally, NC-balloons of different figures were selected to expand the stents under high pressure (20–24 atm) **(d)**, and angiography showed that the stents were fully expanded with a blood flow of TIMI 3 **(e,f)**. **Case 5**: An example of PCI using the GGEC I in CTO of the RCA with abnormal opening. Angiography showed that the ascending aorta was significantly widened, the end of RCA 2 segment showed localized occlusion, and the bridging collaterals supplied blood to make the distal vessels partially visible **(a)**. The LAD provided the collaterals, and the RCA was retrogradely perfused to the end of segment 3 **(b)**. It was difficult to keep the 6F AL 1.0 guiding catheter in place, and Sion was patiently manipulated to “float” into the RCA **(c)**. Then, the GGEC I was slowly pushed along Sion into the RCA **(d)**. Sion was exchanged with Conquest pro 8–20 via the Finecross and manipulated carefully through the occluded segment **(e)** and into PL **(f)** under multi-position fluoroscopy. Under the support of the GGEC I, pre-expansion was performed using balloons of different specifications **(g,h)**. Finally, the stent was successfully implanted in the occlusive segment **(i)**. **Case 6**: An example of PCI using the GGEC I to release the burr incarceration. 6F EBU 3.75 angiography showed heavy calcification in LAD 6-8 segments with about 90% stenosis **(a)**. Runthrough was carefully manipulated to reach the end of the LAD through the narrow lesions, and NC Trek 2.0 × 12 mm was selected for high-pressure dilatation (20–24 atm). However, the balloon was still not fully expanded, and the body had obvious indentation **(b)**. Rotational atherectomy was started and a 1.5-mm burr was passed through the stenosis successfully, but it was incarcerated during the third polishing process **(c)**. The first attempt to insert a second guidewire and dilate the stenosis near the burr with balloon was unsuccessful **(d)**. The rotational catheter was immediately cut off, and the GGEC I was sent into the guiding catheter. It reached the LAD 6 segment along its inner core. After wrapping it tightly with the non-invasive head end of the GGEC I (dotted white line), the burr was successfully removed from the body together with the GGEC I **(e)**. Finally, stents were successfully implanted after NC-balloon dilation **(f)**.

There were 237 target lesions treated, with the majority being type C lesions (95.8%), followed by B2 lesions (4.2%). Among the included cases, there were 104 cases of right coronary artery (RCA; 43.9%), 76 of left ascending artery (LAD; 32.0%), 40 of left circumflex artery (LCX; 16.9%), and 17 of LMCAD (7.2%). The most common indication for GGEC I use was heavy calcification (67%), followed by extreme tortuosity (12.2%), extreme tortuosity and heavy calcification (10.9%), distally located lesion (4.5%), picking up the retrograde wire when using the active greeting technique (AGT; 3.2%), anomalous origin of the vessel (1.8%), and releasing the burr incarceration (0.4%) (Figure 2). The typical examples of using the GGEC I during TRI in different situations are illustrated in Figure 3.

**FIGURE 4:**
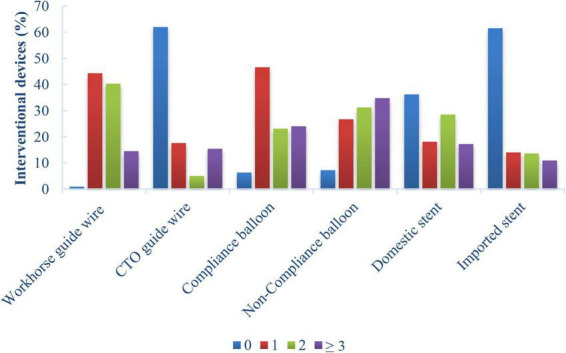
Percentages of interventional devices in patients who underwent TRI using the GGEC I (*n* = 221).

**FIGURE 5:**
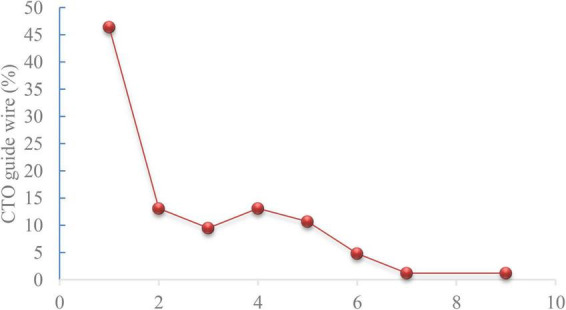
Chronic total occlusion (CTO) guide wire usage analysis (*n* = 84).

In most cases, the GGEC I was inserted more than 20 mm deep into the vessels using the balloon-assisted sliding and tracking (BLAST) technique. In 18 cases (7.6%), TRI was successfully completed with the help of GGEC I after rotational atherectomy. The mean operative time was 58 minutes, and the overall success rate was 94.1%, with four patients receiving DCB treatment without stents. For each vessel, CTO had a longer operative time (RCA 93 *vs.* 63.5 min; LAD 76.5 *vs.* 53.5 min; LCX 64 *vs.* 57.5 min; *P* < 0.05) and a lower success rate (RCA 88.2% *vs.* 92.3%; LAD 85% *vs.* 96.1%; LCX 84.6% *vs.* 92.5%; *P* < 0.05) (Table 3). Furthermore, the analysis results for interventional devices showed that one to two workhorse guide wires (44.3 and 40.3%, respectively) were needed in most cases, while one or more than three CTO guide wires (17.6 and 15.4%, respectively) were needed for opening the CTO lesions (Figures 4, 5). On the other hand, one pre-dilated balloon (46.6%) and two or more than three post-dilated balloons (31.2 or 34.8%, respectively) were usually used for lesion modification and optimization, with a high proportion of domestic stent implantation (63.8%) (Figure 4). No GGEC-associated procedural complications (dissection, stent dislodgement, shaft breakage, etc.) were reported. Two patients (0.9%) with LAD PCI experienced subacute stent thrombosis on the fourth and sixth day after stenting and were successfully treated by emergency high-pressure dilation with non-compliant (NC)-balloon. No other procedure-related complications and major clinical events occurred during the hospitalization or follow-up period of at least 1 month.

**TABLE 3:** Procedural data of patients who underwent TRI using GGEC I (*n* = 221).

			Median (minimum – maximum)								
			
Target vessel	Case No *n* (%)	Rotablation *n* (%)	Workhorse guide wire	CTO guide wire	Pre-dilated balloon	Post-dilated balloon	Domestic stent	Imported stent	Length of stent (mm)	Operating time (min)	Success rate *n* (%)
RCA	104 (43.9)	5 (4.8)	1 (1–4)		2 (0–11)	2 (0–6)	1 (0–4)	0 (0–4)	66(0–131)	63.5 (10 – 269)	96 (92.3)
CTO	51 (49.0)			1 (1–7)						93 (10–269) *	45 (88.2) *
LAD	76 (32.1)	9 (11.8)	2 (1–4)		1 (0–5)	2 (0–7)	2 (0–3)	0 (0–3)	59.5(0–104)	53.5 (20 – 396)	73 (96.1)
CTO	20 (26.3)			1 (1–9)						76.5 (39 – 396) *	17 (85) *
LCX	40 (16.9)	1 (2.5)	1.5 (1–3)		1.5 (0–5)	1 (0–5)	1 (0–3)	0 (0–4)	36(0–112)	57.5 (18 – 179)	37 (92.5)
CTO	13 (32.5)			1 (1–3)						64 (37 – 179) *	11 (84.6)*
LM + LAD	15 (6.3)	3 (20)	2 (1–4)		1 (0–4)	3 (1–7)	1.5(0–3)	0.5 (0–3)	61.5(0–96)	79.5 (32 – 396)	14 (93.3)
LM + LCX	1 (0.4)	0	2		1	2	0	4	112	157	1 (100)
LM + LAD + LCX	1 (0.4)	0	2		1	2	0	3	64	111	1 (100)
Total	237 (100)	18 (7.6)	2 (1–4)	2 (1–9)	1 (0–11)	2 (0–7)	1 (0–4)	0 (0–4)	58 (0–131)	58 (10,396)	223 (94.1)

*P < 0.05; RCA, right coronary artery; CTO, chronic total occlusion; LAD, left ascending artery; LCX, left circumflex artery; LM, left main coronary artery.

Stratification based on the SYNTAX I score showed that there were no significant differences in procedure time and success rate among low (<23), intermediate (23–32), and severe (>32) CAD patients treated with TRI using the GGEC I (*P* > 0.05). The procedure times were 58.5, 54, and 57 min, respectively, while the success rates were 95.4, 93, and 93.7%, respectively (Table 4).

**TABLE 4:** Analysis of failure cases who underwent TRI using GGEC I according to SYNTAX I Score (*n* = 221).

SYNTAX I Score	Failure cases No	Gender	Age (years)	Target vessel	Workhorse guidewire	CTO guidewire	Through the occluded lesion (Yes/No)	Compliance balloon (*n*)	Operating time (min)
<22	1	M	54	LCX CTO	1	3	Yes	4	77
Total procedure time (min)*	58.5 (18–126)								
Total success rate, *n* (%)	95.4%								
23–32	1	F	51	LCX	2	/	/	3	59
	2	M	69	LAD CTO	1	3	Yes	4	60
	3	M	57	RCA CTO	2	4	Yes	1	105
	4	M	74	RCA CTO	1	1	No	0	84
Total procedure time (min)*	54 (20–269)								
Total success rate, *n* (%)	93%								
≥33	1	M	66	LCX CTO	1	1	Yes	2	38
	2	M	74	RCA CTO	1	0	No	0	10
	3	M	72	RCA	4	/	/	2	67
	4	M	82	LAD CTO	3	4	Yes	2	135
	5	M	74	RCA	1	/	/	2	30
	6	M	67	LAD CTO	3	2	Yes	1	82
	7	M	56	RCA CTO	3	2	Yes	4	230
	8	M	67	RCA CTO	2	6	Yes	1	158
	9	M	72	RCA CTO	2	2	Yes	2	114
Total procedure time (min)*	57 (10–396)								
Total success rate, *n* (%)	93.7%								

*Median (minimum – maximum); RCA, right coronary artery; CTO, chronic total occlusion; LAD, left ascending artery; LCX, left circumflex artery.

In this study, 14 cases were reported as failure cases. In the low category, the only failure case was a 54-year-old male patient with LCX CTO as the target lesion. During the procedure, although the guidewire successfully passed through the occlusion and entered the distal true lumen, the pre-dilated balloon of 1.5 mm or 1.25 mm in diameter could not pass through the occluded lesion even after repeated attempts at providing support with the GGEC I due to extreme vascular tortuosity and heavy calcification; consequently, the operation was terminated. In the intermediate group, four operations failed, including the case of a 74-year-old male patient with RCA CTO as the target lesion, as the guidewire failed to pass through the occluded lesion. In the remaining three patients with LAD CTO, RCA CTO, and LCX as the target lesion, respectively, the reason for failure was extreme vascular tortuosity and heavy calcification preventing the small-sized pre-dilated balloon from passing through or effectively dilating the target lesion even with the support from the GGEC I. In the high severity group, a total of nine failed cases (all males) were reported, including seven cases with CTO lesions (one LCX, two LAD, and four RCA cases). Except for a 74-year-old male patient with RCA CTO who suddenly changed his mind and refused the operation, although the guidewire successfully passed through the occluded lesions, in the remaining six CTO and two RCA cases, the operation was stopped as the small-sized pre-dilated balloon could not pass through or effectively dilate the target lesion, even with the GGEC I’s support owing to extreme vascular tortuosity and heavy calcification (Table 4).

## Discussion

The present study suggested that the GGEC I used in TRI could be beneficial, with few complications. The target vessel’s anatomical characteristics might be the determinants of GGEC I use, regardless of the SYNTAX I or II score, contributing to increasing the success rate of complex PCI in patients with CAD.

In the present study, the overall mean operation time was 58 min, and the overall success rate was 94.1%. A roughly similar success rate was reported in a previous study (12). Expectedly, significantly longer operative time and significantly lower success rate were observed for the CTO compared with the corresponding vessels (21). The current results indicate that the CTO remains one of the greatest challenges faced by interventional cardiologists. Thus, a hybrid strategy should be actively implemented to open complex coronary CTO efficiently, safely, and precisely. In addition, one to two workhorse guidewires were required for most cases, while one or two to four CTO guidewires were used for opening the CTO. This usage was mainly dependent on the interventional cardiologist’s knowledge of the anatomical characteristics of the CTO, the understanding of the CTO guidewire, and their ability to manipulate the CTO guidewire (22).

Furthermore, in most cases, more than two NC-balloons (66%, *n* = 221) and domestic stents (63.8%, *n* = 221) were used. The implementation of plaque modification of tortuous and calcified lesions (23–26) and the optimization of stent implantation (27, 28) might have contributed significantly to the relatively good short-term prognosis in this study. Of course, it is also dependent on the interventional cardiologist’s experience and operation skills (22). Notably, this study detected two patients (0.9%) with the culprit anterior descending branch who developed subacute thrombosis within 1 week of the PCI due to stent malapposition caused by heavy vascular calcification. They were treated by an emergency procedure of NC-balloon high-pressure dilation. No other major cardiovascular events were reported during the observation period in the hospital or during a 1-month follow-up after discharge. Therefore, the GGEC I is probably safe in TRI even in CAD patients with a high-risk SYNTAX score; in addition, a favorable long-term outcome is expected for the patients who underwent successful PCI.

The GGEC I is a very useful tool in challenging cases of complex PCI, as its increased intubation depth provides a stronger backup force (11, 12, 29). Nevertheless, it should be noted that, as per the manufacturer’s instructions, the extension of the catheter from the guiding catheter should be less than 15 cm. Otherwise, the mother-in-child catheter might lose coaxiality and hinder the withdrawal of the interventional device. In addition, it should be noted that the blunt tip with edged tubular structure of the GGEC I may be caught in fibrous plaques or stent metal mesh beams while pushing along the diseased coronary artery. On the other hand, the edge of the stainless-steel collar may hinder the interventional devices into the GGEC I. Therefore, non-standard procedures can lead to a series of perioperative complications, including shaft breakage, stent stripping or dislodgement, coronary artery dissection, and even perforation, incarceration, etc., and should especially be considered by inexperienced interventional cardiologists while treating patients. Compared with first-generation guide extension systems, including the GuideLiner™ (Teleflex, Morrisville, NC, United States), the GuideZilla™ (Boston Scientific, Natick, MS, United States) and the Telescope™ (Medtronic, Santa Rosa, CA, United States), the CrossLiner™ with a leading, flexible, low profile, monorail and inner microcatheter (0.017” leading “microcatheter” tip) may overcome the deficiencies of “blunt-end” tubular structures and allow safe, deep, coronary intubation (30), although further clinical validation is needed.

Based on the reported cases and previous studies (10, 11, 13), the following techniques were summarized. The GGEC I should be pushed into the guiding catheter through the Y-connector along the guidewire in the same direction and at a constant slow speed. When the GGEC I is difficult to be pushed through the lesion, the lesions must first be elaborately modified with multiple pre-dilatations (even with NC-balloons) along the way before trying to push once more. It can also be carefully and slowly pushed forward close to the target lesion site under fluoroscopy with the help of a dual guide wire support if necessary. Of course, the most desirable method is to use the BLAST technique in this situation. During the procedure, complications such as coronary artery injury, dissection, hematoma expansion, and longitudinal compression of the implanted stent should be monitored. When it is difficult to enter the guidewire, balloon, stent, or other interventional devices into the GGEC I due to resistance, which usually occurs when the aortic arch is extremely tortuous, the devices should be slightly retracted and rotated to adjust the angle, and then re-inserted. On the other hand, the abovementioned interventional devices can be successfully inserted into the guiding catheter of the GGEC I after an 8–10-atm dilation of the edge of the stainless-steel collar with a pre-dilation balloon with a diameter of 2.0 mm. The GGEC I should be promptly withdrawn into the guiding catheter or the normal vascular segment after the balloon or stent is placed on the target lesion to avoid interference with coronary blood flow. Although the 6F GGEC I has a large inner diameter (0.057 inches), enabling the delivery of most interventional devices, it cannot be sent into the covered stent (0.064–0.068 in).

At present, the main indications for GGEC I use are: (1) anomalous origin or angulated take-off of native coronary arteries; (2) extremely tortuous vessel; (3) heavy calcification; (4) distally located lesion; (5) picking up the retrograde wire during CTO intervention; (6) releasing the burr incarceration (11, 30, 31). In the present study, the main indications were heavy calcification (67%) followed by extreme tortuosity (12.2%), extreme tortuosity and heavy calcification (10.9%), distally located lesion (4.5%), picking up the retrograde wire when using the active greeting technique (AGT, 3.2%), anomalous origin of the vessel (1.8%), and to release the burr incarceration (0.4%). In addition, the GGEC I is usually used for remedial purposes, which inevitably leads to longer operation time, higher radiation exposure dose, and higher contrast agent dosage (11, 31, 32). Still, the results of the SYNTAX I score stratification in this study indicate that the SYNTAX score was beneficial in guiding the revascularization strategy, and anatomical characteristics were more significant in the successful treatment of a single target vessel.

Based on the observations mentioned above, there is reason to suggest that the anatomical characteristics of target coronary artery lesions can be quantified as follows. Diffuse, tortuous, calcification, angulation, CTO, abnormal coronary openings, and distal lesions can be scored as 1, 3, 3, 3, 2, 1, and 1, respectively. With an accumulated score ≥ 3, especially for patients belonging to the intermediate (23–32) and severe (>32) CAD groups, which are based on the SYNTAX I score, the GGEC I should be used actively to achieve a successful result. Its usage might shorten the operative time, reduce the radiation exposure dose, decrease the amount of contrast agent and avoid the risk of possible complications such as contrast-induced nephropathy (CIN) (12). Still, these possible advantages need to be confirmed in large randomized controlled trials. In addition, in the process of rotational atherectomy, the GGEC I can be used to release the burr incarceration, which is the best emergency treatment method (as shown in Figure 3, case 6). It could help avoid the fatal complications caused by long-time incarceration or the burr lodged in the vessel. The GGEC I can be prepared in advance for complex rotational atherectomies, including extreme tortuosity, calcification, and angular lesions.

This study had several limitations. It had a retrospective, single-center study design. The performance of the intervention might be influenced by the cardiologist’s skills and experience. Therefore, to address these limitations, a randomized controlled trial should be conducted based on the anatomical characteristics of the target coronary artery lesions.

In conclusion, the GGEC I might be beneficial, with few complications for complex PCI (TRI), and is not affected by SYNTAX score stratification. The anatomical properties of the target vessel might be the determinant for GGEC I use. Therefore, interventional cardiologists should predict the procedural difficulties and actively use this tool to achieve successful PCI, especially in the intermediate and complex cases based on the SYNTAX score.

## Data availability statement

The original contributions presented in the study are included in the article/supplementary material, further inquiries can be directed to the corresponding author/s.

## Ethics statement

The studies involving human participants were reviewed and approved by the Ethics Committee for Human Study of the First Affiliated Hospital of Xi’an Jiaotong University (XJLS-2016-354). The study was conducted in compliance with the ethical principles of the Declaration of Helsinki. The requirement for informed consent was waived by the committee. Written informed consent for participation was not required for this study in accordance with the national legislation and the institutional requirements.

## Author contributions

XL conceived the clinical study and made substantial contributions to the study design and data acquisition, analysis, and interpretation, as well as drafted the manuscript. QL, YF, YX, and DW participated in designing and conducting the study and interpreting the results. MD, JL, and TY oversaw the data collection and input processes. All authors contributed to revised the manuscript and gave final approval for manuscript publication.
